# Mitral transcatheter edge-to-edge repair with inverse leaflet grasping in the setting of prior atrial septal defect closure: a case report

**DOI:** 10.1093/ehjcr/ytag187

**Published:** 2026-03-10

**Authors:** Andreas Ochs, Nicolas Geis, Matthias Eden, Norbert Frey, Mathias H Konstandin

**Affiliations:** Department of Cardiology, Angiology and Pneumology, University Hospital Heidelberg, Im Neuenheimer Feld 410, Heidelberg 69120, Germany; DZHK (German Center for Cardiovascular Research), partner site Heidelberg/Mannheim, Germany; Department of Cardiology, Angiology and Pneumology, University Hospital Heidelberg, Im Neuenheimer Feld 410, Heidelberg 69120, Germany; DZHK (German Center for Cardiovascular Research), partner site Heidelberg/Mannheim, Germany; Department of Cardiology, Angiology and Pneumology, University Hospital Heidelberg, Im Neuenheimer Feld 410, Heidelberg 69120, Germany; DZHK (German Center for Cardiovascular Research), partner site Heidelberg/Mannheim, Germany; Department of Cardiology, Angiology and Pneumology, University Hospital Heidelberg, Im Neuenheimer Feld 410, Heidelberg 69120, Germany; DZHK (German Center for Cardiovascular Research), partner site Heidelberg/Mannheim, Germany; Department of Cardiology, Angiology and Pneumology, University Hospital Heidelberg, Im Neuenheimer Feld 410, Heidelberg 69120, Germany; DZHK (German Center for Cardiovascular Research), partner site Heidelberg/Mannheim, Germany

**Keywords:** Mitral transcatheter edge-to-edge repair, M-TEER, Mitral regurgitation, PASCAL, Case report, Inverse leaflet grasping

## Abstract

**Background:**

According to the 2025 European Society of Cardiology (ESC)/European Association for Cardio-Thoracic Surgery guidelines, transcatheter edge-to-edge repair has a Class IIa recommendation in patients with primary mitral regurgitation at high surgical risk following Heart Team evaluation. Procedural complexity may be increased by prior septal closure devices and challenging leaflet anatomy. We describe a modified technical approach that enabled successful treatment in this setting.

**Case summary:**

A 75-year-old woman with a history of atrial septal defect closure presented with advanced heart failure symptoms due to severe primary mitral regurgitation from posterior leaflet prolapse. Because of frailty and comorbidities, surgical repair was not feasible, and an interventional approach was recommended. Transseptal puncture was technically challenging due to an Amplatzer occluder and required a posterior–superior puncture site. Conventional leaflet capturing with the PASCAL Ace device was unsuccessful because of pronounced prolapse and localized chordal restriction. A modified sequence, called inverse leaflet grasping, was employed: clasps were fixed in a horizontal position, while the paddles remained elongated during posterior leaflet engagement, followed by paddle closure. This manoeuvre allowed secure capture and effective reduction of regurgitation after the first device, followed by implantation of a second device using conventional technique. The patient improved clinically and remained stable at 6-month follow-up with mild residual mitral regurgitation.

**Discussion:**

This case illustrates that mitral transcatheter edge-to-edge repair is feasible even in anatomically complex scenarios. Inverse leaflet grasping may serve as a bailout technique when conventional leaflet capturing fails. The report underlines the importance of tailored interventional strategies in line with current ESC guidelines.

Learning points

- To understand the challenges of transseptal access for mitral transcatheter edge-to-edge repair in patients with prior atrial septal defect closure.
- To recognize inverse leaflet grasping as a potential bailout strategy when conventional leaflet grasping fails in severe mitral valve prolapse.
- To appreciate the importance of guideline-directed Heart Team decision-making in high-risk patients with primary mitral regurgitation.

## Introduction

Mitral transcatheter edge-to-edge repair (M-TEER) has become an established treatment option for patients with severe primary mitral regurgitation (MR) who are at high surgical risk or considered inoperable after Heart Team evaluation.^1^ While procedural success rates are generally high, certain anatomical and technical factors can substantially increase procedural complexity. Among these, the presence of prior atrial septal defect (ASD) closure devices may limit transseptal access, and complex leaflet pathology, such as severe prolapse or chordal restriction, can impair effective leaflet capturing. In such challenging settings, technical adaptations may be required to achieve procedural success.

We report the case of an elderly patient with severe degenerative MR with a history of ASD closure. Conventional leaflet capturing with the PASCAL Ace device was unsuccessful; however, a modified strategy—inverse leaflet grasping—enabled secure posterior leaflet engagement and effective reduction of MR.

## Summary figure

**Figure:**
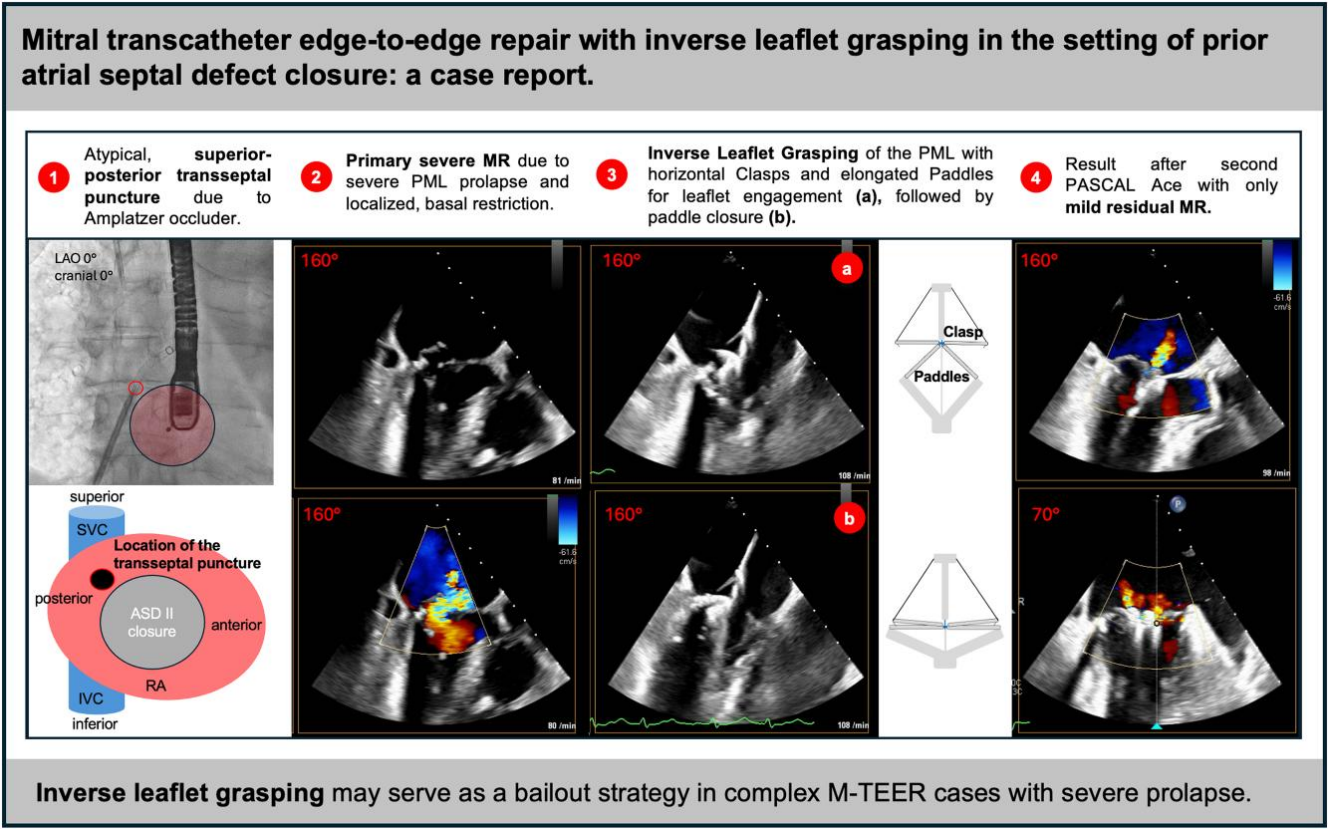


## Case presentation

In 2002, the patient was diagnosed with an ASD Type II and a left-to-right shunt of 55%, which was closed interventionally with an Amplatzer device (24 mm) due to exertional dyspnoea. The procedure was uncomplicated, and she remained clinically stable for many years.

In May 2024, at the age of 75 years, she was admitted to a non-tertiary hospital with acute decompensated heart failure. Transthoracic echocardiography revealed severe eccentric MR with preserved left and right ventricular systolic function, severe diastolic dysfunction, elevated pulmonary artery systolic pressure (50 mmHg), and marked left atrial dilation. Mild-to-moderate functional tricuspid regurgitation was present, and the aortic valve showed sclerotic changes without significant stenosis. The underlying mitral valve pathology was further assessed by transoesophageal echocardiography (TEE), which confirmed posterior mitral leaflet (PML) prolapse with localized restriction. However, TEE image quality was limited, and some aspects of the pathology were not fully appreciated pre-procedurally. Coronary artery disease was excluded by invasive angiography. Right heart catheterization demonstrated elevated left-sided filling pressures with a mean pulmonary capillary wedge pressure of 25 mmHg and a prominent v-wave of 39 mmHg.

Comorbidities included persistent atrial fibrillation (anticoagulated with apixaban, 5 mg twice daily), arterial hypertension, prior pulmonary embolism (in 2000), and breast cancer treated with surgery, chemotherapy, and radiotherapy in 2015. She was on guideline-directed medical therapy, including ramipril, spironolactone, torasemide, dapagliflozin, and bisoprolol.

Despite this, she reported progressive dyspnoea corresponding to New York Heart Association (NYHA) Class III–IV and orthopnoea requiring her to sleep upright. The patient was referred for surgical repair of primary MR. Although chronologically younger than typical TEER cohorts, her clinical status suggested advanced frailty. After assessment by the interdisciplinary Heart Team, surgical repair was considered not feasible due to frailty and comorbidities, and M-TEER was recommended in line with European Society of Cardiology (ESC) guideline-directed decision-making.

One week before the scheduled procedure, she presented with worsening dyspnoea at rest, anorexia, nausea, and unintentional weight loss of 5 kg over 4 weeks. Clinical examination revealed pulmonary crackles and peripheral oedema. Laboratory testing demonstrated elevated N-terminal pro B-type natriuretic peptide (NT-proBNP) (4161 pg/mL) with normal troponin T. Chest X-ray confirmed pulmonary congestion. She received intravenous loop diuretics and underwent the intervention on Day 5 of hospitalization, approximately 6 weeks after the initial decompensation.

**Figure 1:**
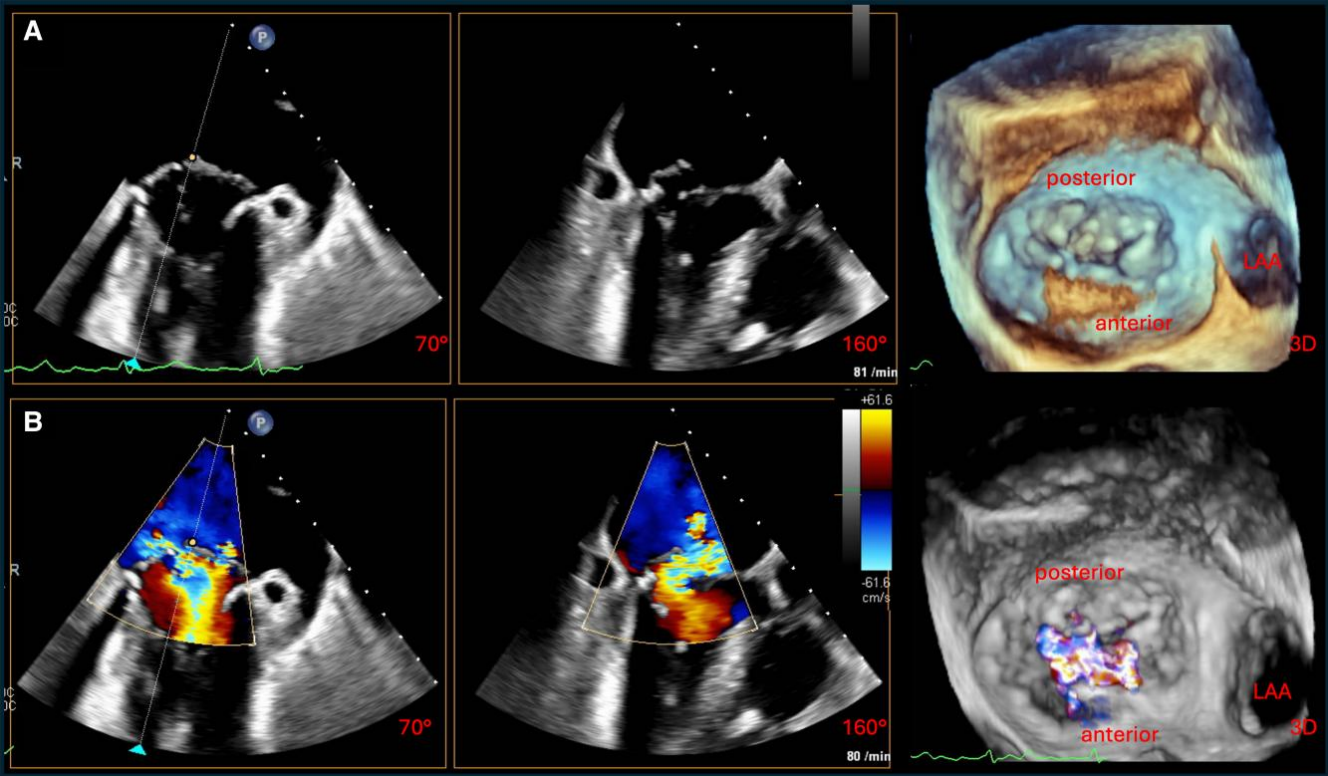
Periprocedural transoesophageal echocardiographic assessment of mitral valve pathology. (*A*) Biplane TEE images (left to right: intercommissural, LVOT, and 3D en face view from the left atrium) without colour Doppler, revealing a pronounced prolapse of the PML with minimal calcification and localized chordal restriction. (*B*) Corresponding views with colour Doppler, demonstrating severe MR due to a combination of leaflet prolapse and tethering, consistent with degenerative mitral valve disease. TEE, transoesophageal echocardiography; LVOT, left ventricular outflow tract; PML, posterior mitral leaflet; MR, mitral regurgitation; LAA, left atrial appendage.

Under general anaesthesia, comprehensive TEE confirmed severe MR due to a pronounced PML prolapse with localized basal restriction and minimal calcification, consistent with mixed degenerative disease (*Figure 1*; Supplementary material online, *Video S1*). Quantitative assessment revealed an effective regurgitant orifice area of 0.83 cm^2^ and a regurgitant volume of 84 mL, with preserved valve area (4.3 cm^2^). The pre-procedural imaging underestimated chordal restriction, increasing procedural complexity.

**Figure 2:**
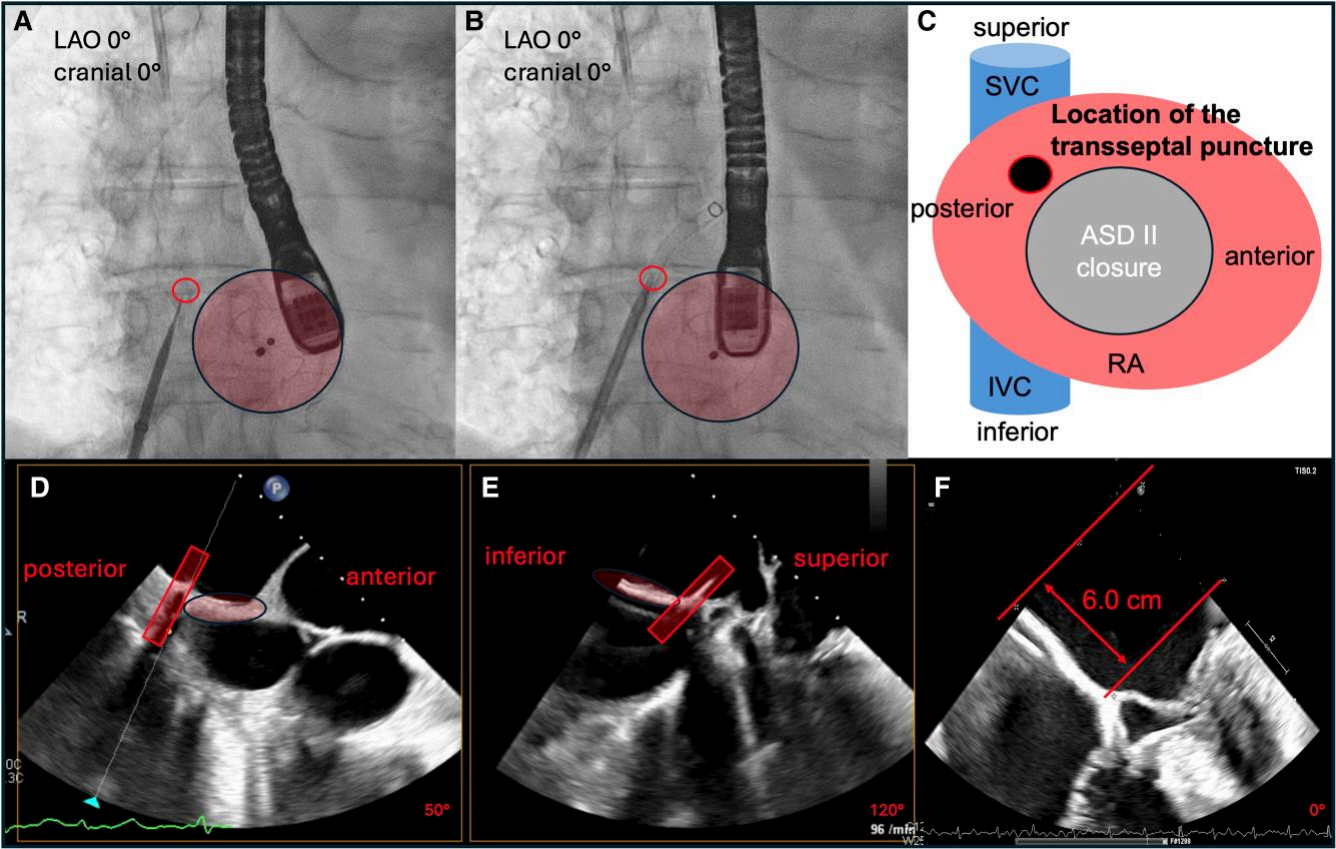
Imaging of the transseptal puncture procedure. (*A* and *B*) Fluoroscopic views demonstrating the technically challenging transseptal puncture in the presence of the existing Amplatzer septal occluder device. (*C*) Schematic illustration of the interatrial septum viewed from the right atrium, showing the SVC and IVC and the location of the transseptal puncture in relation to the Amplatzer device. (*D* and *E*) TEE images showing the position of the transseptal puncture site (highlighted by a rectangle) in relation to the Amplatzer device (marked by an ellipse). (*F*) TEE image depicting the resulting puncture height (∼6 cm) relative to the mitral valve plane. LAO, left anterior oblique; SVC, superior vena cava; IVC, inferior vena cava; TEE, transoesophageal echocardiography; RA, right atrium.

Transseptal access was technically demanding due to the prior implantation of the Amplatzer device. A posterior–superior puncture site was achieved under TEE and fluoroscopic guidance, yielding a working height of ∼6 cm above the mitral annulus (*Figure 2*).

**Figure 3:**
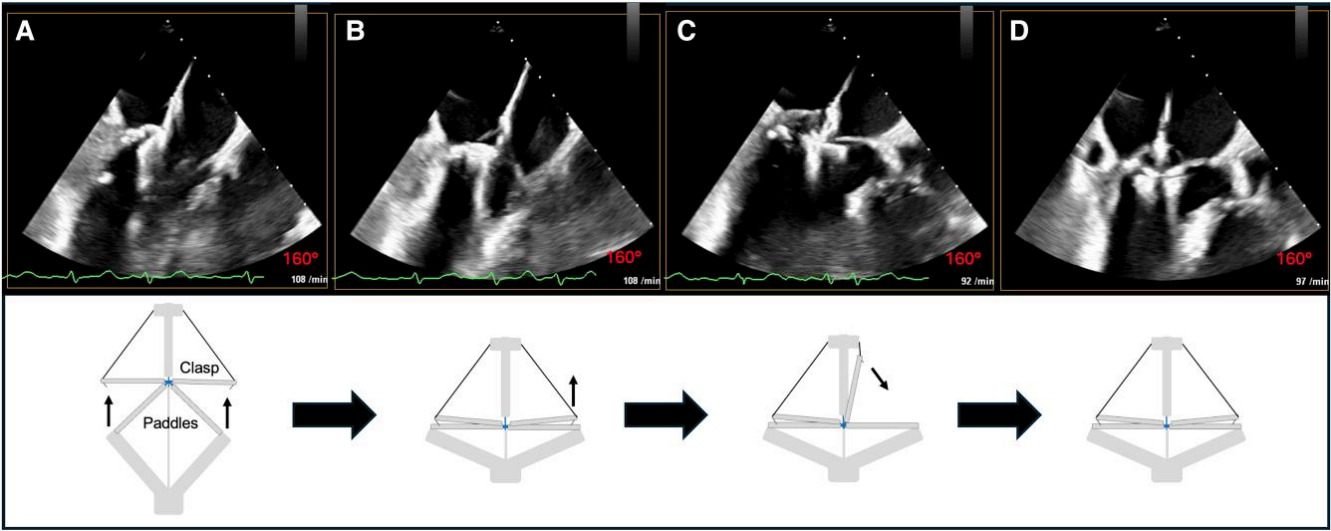
‘Inverse leaflet grasping’ technique for PML engagement and anterior leaflet grasping using the PASCAL Ace system. (A–D) In each panel, the upper image shows the TEE LVOT view (160°), while the lower image, a schematic illustration of device configuration. (*A*) Initial device positioning with clasps in horizontal position and paddles in elongated configuration. The prolapsing PML is engaged in this configuration. (*B*) Gradual closure by lowering the paddles into a horizontal position, securing the PML and completing inverse leaflet grasping. (*C*) Opening of the anterior clasp for conventional capture of the AML, with the AML already engaged. (*D*) Both clasps lowered, demonstrating successful capture of the PML (inverse technique) and AML (conventional technique). TEE, transoesophageal echocardiography; LVOT, left ventricular outflow tract; PML, posterior mitral leaflet; AML, anterior mitral leaflet.

A PASCAL Ace device (Edwards Lifesciences, Irvine, California, USA) was positioned centrally using the PASCAL precision system. Standard leaflet capture was attempted with horizontally aligned paddles and both simultaneous and sequential grasping with the clasps. However, severe prolapse and restricted basal chordae prevented stable engagement. To overcome this, a modified sequence called inverse leaflet grasping was employed: the clasps were oriented horizontally, while the paddles remained elongated to engage the prolapsing PML. Once the leaflet tissue was immobilized, the paddles were closed into horizontal alignment, locking the leaflet in place (*Figure 3*; Supplementary material online, *Video S2A*). The anterior mitral leaflet was then captured conventionally (*Figure 3*; Supplementary material online, *Video S2B*).

**Figure 4:**
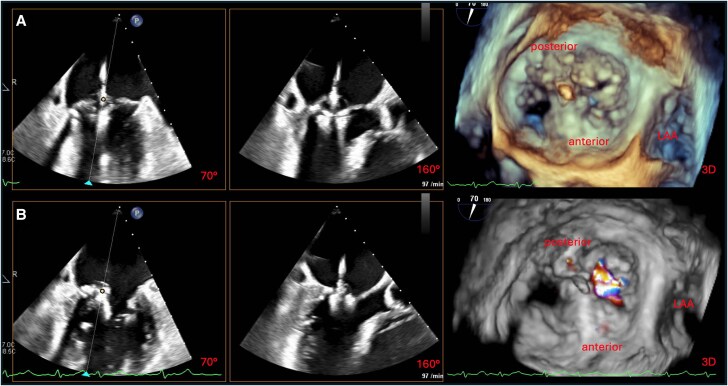
Result after implantation of the first PASCAL Ace device. (*A*) Intercommissural (70°) and LVOT (160°) TEE views showing the device still open after successful engagement of the PML using the inverse technique and the AML using conventional capture. (*B*) Intercommissural (70°) and LVOT (160°) TEE views after device closure, demonstrating effective leaflet approximation. On the right, 3D en face views of the mitral valve without (top) and with (bottom) colour Doppler confirm correct device position and show only a small residual lateral regurgitant jet. LVOT, left ventricular outflow tract; PML, posterior mitral leaflet; AML, anterior mitral leaflet; TEE, transoesophageal echocardiography; LAA, left atrial appendage.

**Figure 5:**
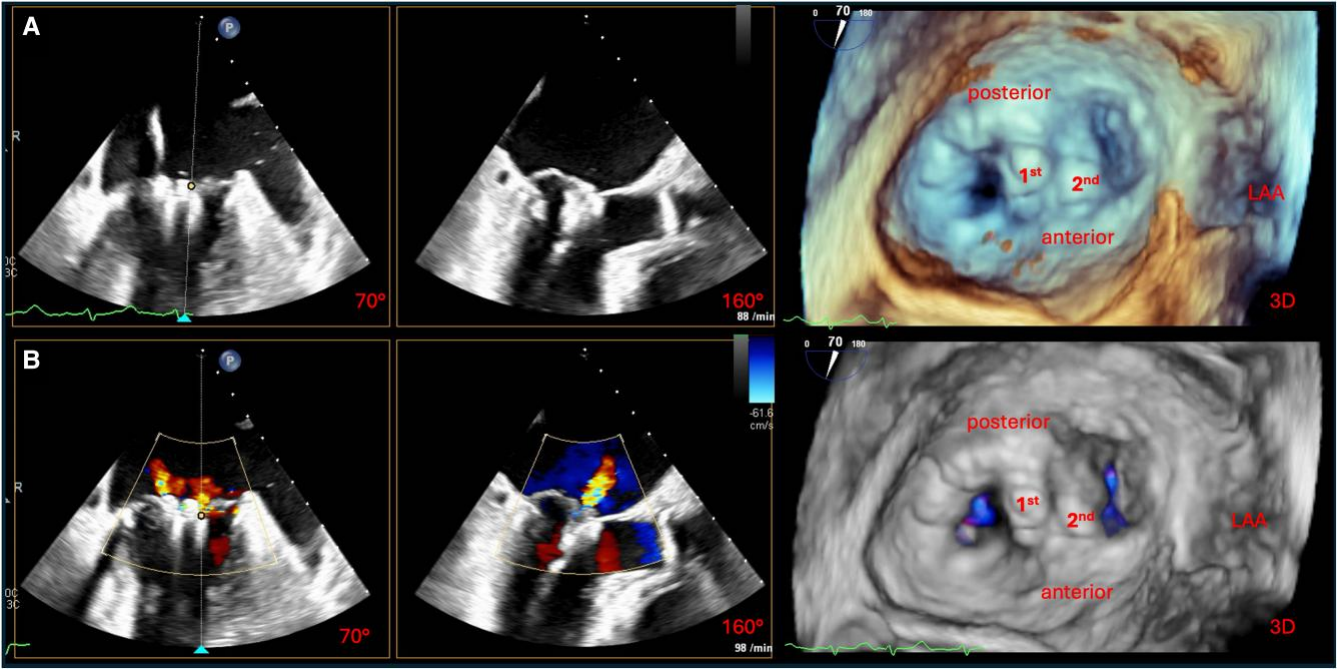
Final result following sequential PASCAL Ace implantation. (*A*) Intercommissural, LVOT, and 3D view without colour Doppler, demonstrating both deployed PASCAL Ace devices in final position. The second device was placed lateral to the first, after successful conventional leaflet capture. (*B*) Corresponding views with colour Doppler, showing only minor residual MR and an overall favourable haemodynamic result with adequate valve area and acceptable transvalvular gradient. LVOT, left ventricular outflow tract; MR, mitral regurgitation; LAA, left atrial appendage.

This manoeuvre achieved significant MR reduction without increasing transmitral gradient (*Figure 4*). A second PASCAL Ace was implanted lateral to the first applying the conventional approach (see Supplementary material online, *Video S3A*), with further reduction of regurgitation. Final assessment showed only mild residual MR and an acceptable mean gradient of 4 mmHg (*Figure 5*; Supplementary material online, *Video S3B*). A small left-to-right shunt at the puncture site of the interatrial septum was left untreated.

The patient experienced immediate symptomatic relief, with marked improvement in dyspnoea. Transthoracic echocardiography performed the following day confirmed stable device position, mild residual MR, and a pulmonary artery pressure of 50 mmHg. Anticoagulation and heart failure therapy were continued, except for ramipril due to hypotension.

At follow-up, the patient remained free from hospitalizations. Dyspnoea persisted on exertion (NYHA III), but oedema had resolved. Echocardiography demonstrated stable device position, preserved biventricular systolic function, and persistent mild MR. Pulmonary artery pressure remained elevated, and NT-proBNP increased compared with baseline, without evidence of device-related obstruction. Overall, symptoms persisted despite adequate valve haemodynamics, consistent with heart failure with preserved ejection fraction; a high daily fluid intake may have contributed to intermittent congestion, and guideline-directed medical therapy was intensified, including initiation of therapy with a sodium–glucose cotransporter 2 inhibitor.^2^

## Discussion

This case illustrates a technically challenging M-TEER in a patient with severe primary MR and prior ASD closure, in whom both atypical transseptal access and posterior leaflet capture were complex. Inverse leaflet grasping enabled effective MR reduction in this setting.

According to current ESC guidelines on valvular heart disease, M-TEER has a Class IIa recommendation for patients with primary MR at high surgical risk following Heart Team evaluation.^1^ In this case, the patient was initially referred for surgery due to her relatively young chronological age. However, frailty and advanced diastolic dysfunction meant that surgical repair was not considered feasible, and the guideline-directed Heart Team evaluation led to the decision for an interventional approach.

The safety and efficacy of M-TEER in high-risk populations have been demonstrated in randomized trials and large real-world registries.^3–5^ The PASCAL system has added technical versatility that may offer an advantage in complex leaflet anatomy.^6,7^ In this patient, reversing the conventional sequence of clasp and paddle deployment allowed for a more controlled engagement of the prolapsing leaflet. This manoeuvre may be valuable when standard capturing fails due to excessive billowing or localized tethering. However, inverse leaflet grasping may be associated with specific risks, including suboptimal leaflet engagement, leaflet injury, or increased procedural complexity due to additional device manipulation. Therefore, this technique should be reserved as a bailout option and performed with careful imaging guidance.

While transseptal access after ASD closure and modified or inverted grasping strategies during M-TEER have been described,^8,9^ the device- and sequence-specific reversal applied in this case, in combination with prior ASD closure, has not been reported to our knowledge. Fallback surgical repair or replacement must remain available in case of procedural failure. Beyond conventional surgery, transcatheter mitral valve replacement is an emerging alternative for high-risk patients with complex degenerative anatomy; however, it is currently limited by anatomical constraints and risks, such as left ventricular outflow tract (LVOT) obstruction and anchoring challenges. In the present case, given feasible repair with durable MR reduction and acceptable gradients, TEER was favoured, but transcatheter mitral valve replacement may become increasingly relevant in the future.^10^

Despite durable reduction of MR, the patient remained symptomatic during follow-up. Post-procedural mean transmitral gradients remained low and within the expected range after TEER, making device-related functional mitral stenosis unlikely. Pre-interventional invasive haemodynamics demonstrated markedly elevated left-sided filling pressures consistent with post-capillary pulmonary hypertension. In the context of long-standing severe primary MR, advanced left ventricular diastolic dysfunction, and persistent atrial fibrillation, these factors likely limited symptomatic improvement despite technical success. Persistently elevated pulmonary artery pressure at follow-up and the overall clinical presentation supported a predominantly heart failure with preserved ejection fraction-related mechanism of persistent symptoms.

## Supplementary Material

ytag187_Supplementary_Data

## Data Availability

The data underlying this article are available within the article and its Supplementary material. Further data are available from the corresponding author upon reasonable request.

## Lead author biography

Andreas Ochs is a cardiologist and researcher at the University Hospitals of Heidelberg and Frankfurt, Germany, with long-standing expertise in cardiovascular imaging, particularly cardiac magnetic resonance (CMR) and cardiac computed tomography (CT). Since 2014, his research has focused on myocardial strain imaging and the clinical utility of fast strain-encoded imaging (fSENC) for non-invasive ischaemia detection. In addition to his research, Dr Ochs has been performing TEE guidance for TEER procedures since 2024. He became a board-certified cardiologist in 2024 and combines his clinical experience in structural heart interventions with advanced cardiac imaging.

## Notes

**Consent:** Written informed consent for submission and publication of this case report, including accompanying images, has been obtained from the patient.
